# Using self and peer video annotations of simulated patient encounters in communication training to facilitate the reflection of communication skills: an implementation study

**DOI:** 10.3205/zma001451

**Published:** 2021-03-15

**Authors:** Anina Pless, Roman Hari, Beate Brem, Ulrich Woermamm, Kai P. Schnabel

**Affiliations:** 1 University of Bern, Institute of Primary Healthcare, Bern, Switzerland; 2 University of Bern, Institute for Medical Education, Bern, Switzerland

**Keywords:** medical education, communication skills training, video, annotation, simulated patients

## Abstract

**Background: **The mandatory communication skills course for fourth-year medical students at the University of Bern Medical School aims to prepare students for challenging communication situations. Students role-play four different scenarios with simulated patients (SPs) and receive feedback from the patient’s perspective. The scenarios are video-recorded and uploaded onto the University’s virtual learning environment. Students can watch and annotate their own videos and give others access to view them.

**Project description:** Although the course is well liked by students, we identified three areas for improvement:

- lack of faculty feedback;
- little active use of the video-recordings;
- lack of opportunity for students to discuss their experiences with each other.

We aimed to address these shortcomings by introducing an additional learning task: students are asked to annotate a section of the video in which they had performed well, and one in which they thought they could have done better, in both their own and a colleague’s videos. These video clips and annotations served as the basis of a subsequent two-hour small-group seminar with a physician tutor. The course was evaluated by a mandatory online questionnaire.

**Results: **All 247 students completed the questionnaire. The annotation tool and task were deemed to be comprehensible. Students believed they had learnt more from annotating a peers’ video than from their own and most thought being assessed by peers was acceptable. The physician tutors’ comments were largely deemed as helpful. The mean mark for the course given by students was 4.6 (median 5) (1=very poor, 6=very good).

**Conclusion: **A communication skills course expanded by video-annotations and group discussions with a physician tutor was shown to be feasible and was well received by students and faculty.

## Zusammenfassung

**Hintergrund:** Der obligatorische Kommunikationskurs für Berner Medizinstudierende im vierten Jahr hat zum Ziel, die Studierenden auf herausfordernde Kommunikationssituationen vorzubereiten. Die Studierenden spielen vier verschiedene Szenarien mit Simulationspatienten (SP) und erhalten von diesen Feedback aus der Patientenperspektive. Die Szenarien werden auf Video aufgezeichnet und auf die virtuelle Lernplattform der Universität geladen. Die Studierenden können ihre eigenen Videos anschauen und annotieren und anderen den Zugang dazu erteilen.

**Projektbeschreibung: **Obschon die Kommunikationskurse bei den Studierenden beliebt sind, identifizierten wir drei Verbesserungsmöglichkeiten:

- Fehlendes ärztliches Feedback
- Spärliche Nutzung der Video-Aufnahmen
- Fehlende Möglichkeit für Erfahrungsaustausch unter den Studierenden

Diesen Defiziten versuchten wir mit einer zusätzlichen Aufgabe entgegenzutreten: Die Studierenden erhielten den Auftrag, von ihren eigenen Videos und denjenigen eines Mitstudierenden jeweils eine gelungene Sequenz, sowie eine in ihren Augen verbesserungswürdige Sequenz zu annotieren. Diese Videosequenzen und Annotationen dienten als Grundlage für ein zweistündiges Kleingruppenseminar mit einem ärztlichen Tutor. Der Kurs wurde mittels eines obligatorischen Online-Fragebogens evaluiert.

**Ergebnisse: **Alle 247 Studierenden füllten den Fragebogen aus. Das Annotations-Tool und die Aufgabenstellung wurden als verständlich beurteilt. Die Studierenden gaben an, mehr von der Annotation des Videos eines Mitstudierenden profitiert zu haben als von der Annotation ihrer eigenen Videos. Die meisten Studierenden störte es nicht, von Mitstudierenden beurteilt zu werden. Die Rückmeldungen durch die ärztlichen Tutoren wurden grösstenteils als hilfreich erachtet. Die Studierenden bewerteten den Kurs durchschnittlich mit der Note 4.6 (Median: 5), (1=sehr schlecht, 6=sehr gut).

**Fazit:** Die Ergänzung des Kurses durch Video-Annotationen und Gruppendiskussionen mit ärztlichen Tutoren konnte erfolgreich implementiert und technisch umgesetzt werden. Der Kurs wurde sowohl von den ärztlichen Tutoren wie auch von den Studierenden gut aufgenommen.

## 1. Background

### Communication skills and reflective practice

Communication skills are a core competency for medical students and physicians. The newly established Swiss learning catalogue PROFILES (Principal Relevant Objectives and a Framework for Integrative Learning and Education in Switzerland) builds on the CanMEDs model, in which one of the medical expert’s main roles is the communicator [1]. 

Simulation is a widely accepted tool to teach communication skills, with evidence of efficacy for transfer into real-life skills [2]. By providing a unique patient view, feedback from SPs during communication skills training enhances both students’ motivation [3] and skills levels [4], [5]. Furthermore, peer-feedback and self-assessment have been shown to be effective in meeting performance goals in communication skills [6], [7]. Both form part of the paradigm of reflective practice, which describes the ability to evaluate an experience critically and thereby develop new understandings for future actions [7], [8]. Reflective practice can be an effective learning strategy in medical education [9], [10]. Using video recordings of clinical performance such as communication situations, which students and peers can access and annotate has been seen to be feasible and was perceived as helpful by students for learning communication skills [11], [12]. Reflection on consultation videos is a powerful tool for learning about microbehaviours (for example head nodding, use of open-ended questions) as well as more holistic skills (for example the use of specific communication skills) [13]. It has shown to have a positive effect on both attitude and performance [14] and has been called the “gold standard of communication skills training” [15]. Feedback on consultation videos allows students to look at themselves from a distance and can lead to “positive self-modelling” if adequately facilitated [13]. Guidance during the reflection process is thought to be essential because of the difficulty in this setting of maintaining the thematic continuity of the discussion [16]. A combination of self-evaluation and feedback from others has been described as having the best effect on clinical performance [17]. 

At the University of Bern Medical School, a communication skills curriculum has been in place for over a decade. The communication skills courses in the fourth year aim to prepare students for challenging communication situations [18]. The topics are motivational interviewing, breaking bad news, informed consent, and taking a sexual history. Students role-play these situations with simulated patients (SPs) and receive feedback from the patient’s perspective as well as from a peer who has observed the encounter. 

Taking into consideration the abovementioned findings in the literature as well as the results of the course evaluations, we decided to revise and expand the communication skills training in the fourth student year. In this paper we explain the original course format and the reasons for making a change, the revised course format, and the implementation process. We also summarise the evaluation results and present an outlook. 

## 2. Project description

### 2.1. Initial format of the communication skills course and rationale for expansion 

The central feature of the fourth-year communication skills course is a series of simulated encounters between individual students and SPs, the latter giving feedback to the students on their performance. 

**Figure 1:**
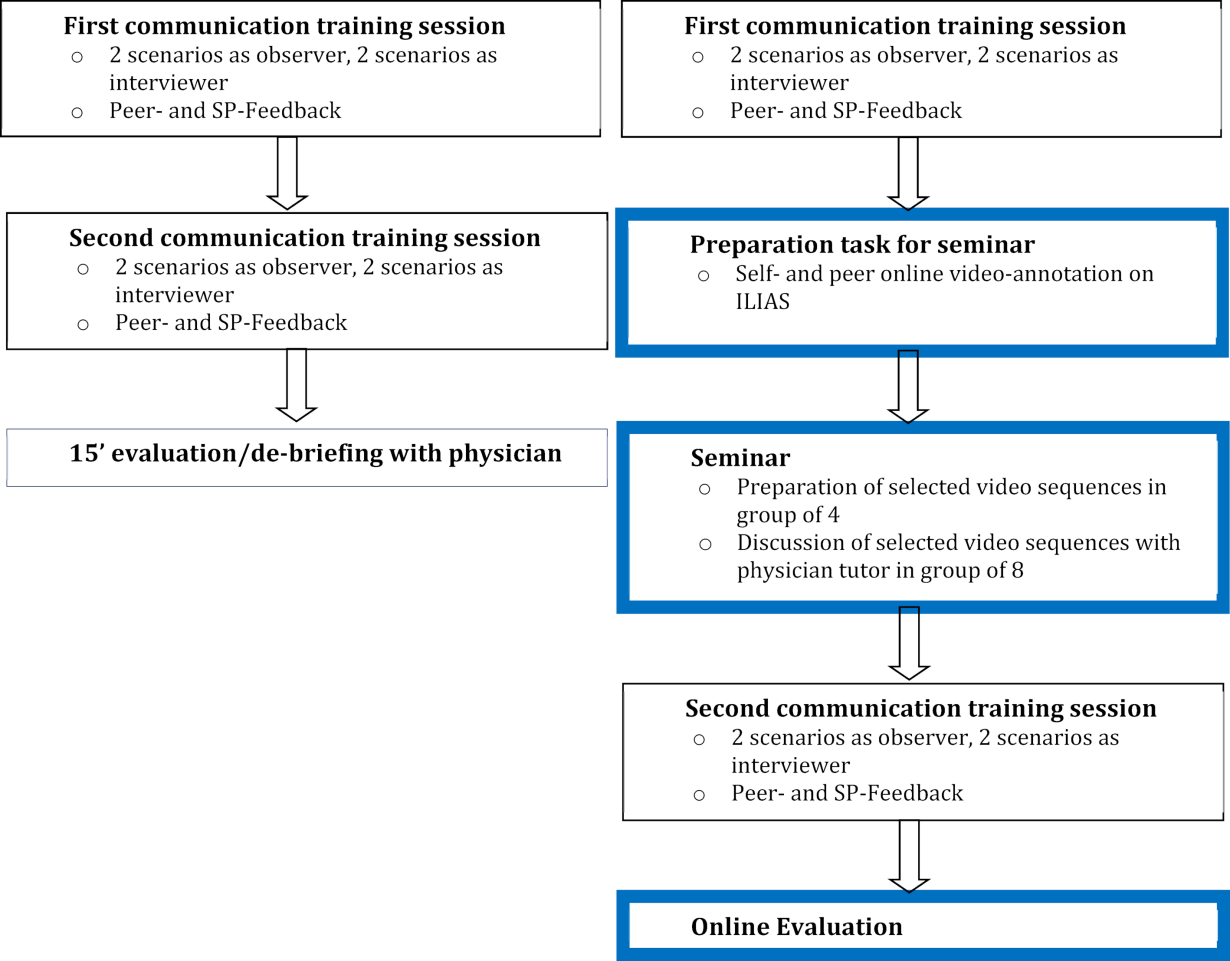
Overview of expanded communication course (newly implemented parts are framed in blue)

In its original format, the course consists of two 2-hour training sessions with SPs. In both training sessions, four scenarios based on the abovementioned topics are enacted with SPs. Students work in pairs, taking it in turns to either play the role of the physician or to observe the other student. A scenario lasts fifteen minutes and is followed by feedback, lasting another fifteen minutes, from the SP as well as from the student observer. An overview of the course, including the new tasks added for this project, is depicted in figure 1 (Fig. 1).

In the second training session the pairs switch roles, each student now playing the role of the physician in the two scenarios they had observed during the first session. Students prepare for the scenarios individually with a detailed study-guide document, which includes background information on the topics, the medical conditions, the Calgary-Cambridge guide [19] and links to online sources [20] for further information about the underlying communication skills models. All training sessions including the feedback sessions are video-recorded and automatically uploaded onto the University’s virtual learning environment. Students can watch and annotate their own videos and give others access to view them.

Course evaluations of students and tutors and user-data of the virtual learning environment yielded three potential areas for improvement:

- Paucity of faculty feedback: in the current setting, there was only a very brief feedback and question session with a physician following the second training session. Time was often too short to discuss students’ questions, for example on how to deal with certain situations in real professional life. Furthermore, the physicians had not witnessed the scenarios, which made giving feedback difficult.
- While all scenarios were video-recorded, only a small percentage of students actually watched their videos after the training sessions and there was no assignment to guide reflection.
- There was no opportunity for students to discuss their experiences with each other and thus learn from each other. Some students said they felt the need to discuss with others what the challenges and positive experiences had been, but there had been no forum for this.

To address these shortcomings and to further improve the communication skills training sessions, we aimed to expand the existing course with a new educational intervention that made use of the self-recorded videos and would foster faculty feedback and peer-to-peer interaction. 

#### 2.2. Expanded course format

**Figure 2:**
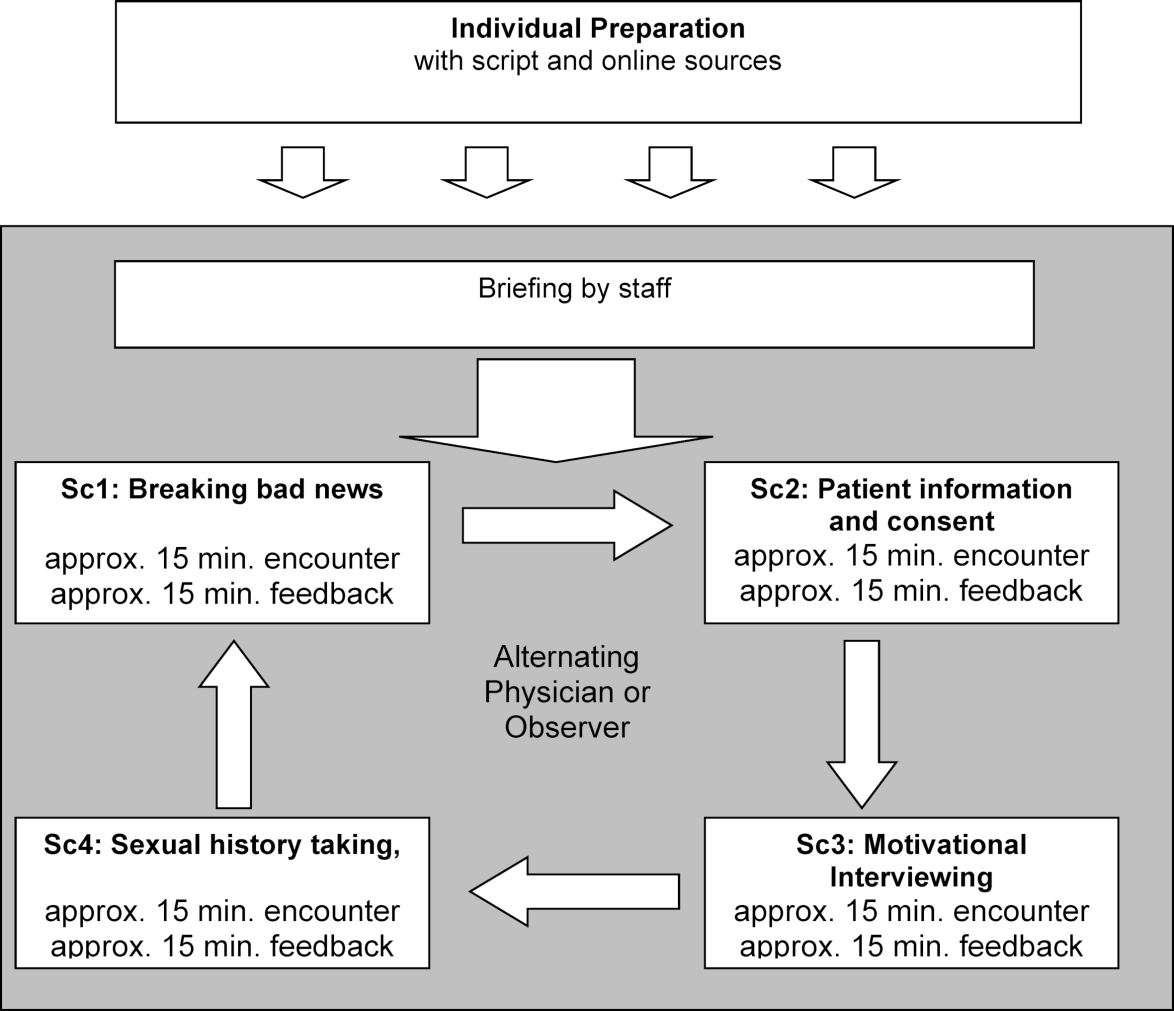
Overview of Communication Skills Training Session (Sc=Scenario)

The basic structure of the two training sessions with SPs was maintained. After the first training session, however, the students were given an online video-annotation task, described below. Students then met in groups of eight to watch and discuss the annotated video clips. These seminars were led by physician-tutors, who also gave input and feedback. The feedback session with physicians that used to take place after the second training was replaced by a mandatory online evaluation of the course, which students could perform immediately after the training (see figure 2 (Fig. 2)).

##### Online video-annotation 

The simulated encounters were video recorded using SWITCHcast [https://www.switch.ch/services/cast/]. The recorded videos were uploaded automatically to the University’s Learning Management System (ILIAS). Since the video recordings were stored in the students’ personal online learning space, the students had to give access to their peers. Each student had the task of watching the videos of their own two scenarios, as well as another student’s video for the same scenarios. The partner of each student was predefined and was not the same as in the training sessions, firstly because every student had already given feedback to their original partner during the training and had heard the SP’s feedback and secondly in order to benefit from watching someone else handling the same scenarios. Each student was asked to label four clips (two of their own, and two of their partner’s) electronically via the online annotation tool , and to write a comment on why they chose these sections, what they found to be good or improvable and why, and make suggestions how this sequence could be improved. The online video annotations formed the basis of the subsequent seminar. On the advice of the University’s legal department, students were given the option to opt-out of recording their encounters. For these students, alternative tasks were available. 

##### Seminar 

The seminars consisted of two parts: 

Part 1: Preparation with peers

Before meeting with the tutor, students were given 45 minutes in groups of four to decide which video clips they wanted to show and discuss during the seminar. In order to have a balanced selection of clips, students were required to choose videos according to the following criteria:

- At least one video clip from each student.
- A balanced number of good and improvable sequences.
- At least two video clips per topic (breaking bad news, etc.).
- No video clip longer than 1 minute.

Students were asked to bring their own mobile devices to present their video clips via the monitors in the seminars.

Part 2: Seminars with physician tutors

Groups of eight students met with a physician tutor for a two-hour seminar immediately after the preparation. The students presented and discussed the video clips that they had chosen. The tutor facilitated the seminars, answered questions, gave feedback and provided information on underlying communication models. At the end of each seminar, students wrote down what their main “take-home” messages were and what they aimed to improve during their second training sessions. 

#### 2.3. Implementation 

The expansion of the existing course was developed, discussed and refined within the management group. We piloted the new concept with sixteen students. This led to the decision to have students do self- as well as peer-annotations and also to introduce the initial 45 minutes for students to prepare their videos before the seminar. The final version of the new format was implemented for all fourth-year students in autumn 2019. We recruited clinically active tutors, and a mandatory two-hour training course familiarised them with the goals and structure of the course. It also allowed them to discuss their role during the seminar, as well as the underlying communication skills concepts of the scenarios. 

## 3. Results

The expanded course format was successfully implemented in the 2019 Autumn term. All 247 students participated in one of the 24 seminars, which took place over the course of eight afternoons. There were no notable problems during the seminars apart from some technical problems with the adapters for the students’ devices, which slowed down the presentation of the selected scenarios in some groups. All students used video-recording, none made use of the exemption rule. 

### 3.1. Online evaluation 

The management group (BB, RH, AP, KS, UW) discussed the areas to be covered in the online evaluation. UW and AP then formulated specific questions, which were then again discussed and refined in the management group until consensus was reached. The evaluation consisted of six questions concerning preparation for the course, seven questions concerning the annotation task and ten questions concerning the seminar. For most questions, we used four-level Likert items (agree, partly agree, partly disagree, disagree) and also provided the option for comments. Some questions required an open-ended response (e.g. “What did you specifically like about the seminar and think should be maintained?” or “What can we improve about the seminar?”). A detailed list of the questions can be found in the attachment 1 . 

All 247 students participated in the online course evaluation. The annotation task was deemed to be easy to understand (88%) and easy to implement (82%) by most students. It took students on average 64 minutes (mean) to complete their online annotations. The majority of students stated that they had benefited from annotating their videos. More students considered watching peers’ videos to be more valuable (66%) than watching their own videos (53%). Some students commented that it was helpful to see another person dealing with the same situation. Other students stated that, while they did not benefit much from annotating someone else’s video, they were able to gain insights from the annotations made by their peers on their own videos. Frequently, students remarked that because the SP and peer feedback during the first training was so detailed, they did not gain any new insights when watching their own videos. However, several students welcomed the opportunity for self-reflection that annotating their own videos provided. The large majority of students (96%) had no objection to their videos being annotated by a peer. 

Most students stated that the small group preparation task was clearly formulated (75%) and that the allocated time for the task was the right length (58%), while some students found the allocated time to have been too long (37%). The majority of students found the discussion in the group with a tutor to have been beneficial (64%) and the tutor’s feedback to have been instructive (67%). The duration of the seminar was deemed to be too long by many (51%). Half of the students (52%) stated that they had been able to apply what they had learned in the second, subsequent training session. Students commented that, because the scenarios of the second training session changed (learners who trained breaking bad news and patient information and consent during the first training session trained motivational Interviewing and sexual history taking during the second session and vice versa), transferring what they had learned in the seminar was often difficult. The group size was perceived to be adequate, as well as the opportunity to share experiences with peers and to benefit from the physician tutor’s experience and perspective. Shorter preparation and seminar time, as well as a shorter time between the training sessions and the seminar, were suggested.

The mean mark given by students to the seminar was 4.6 (median 5) (1=very poor, 6=very good). 

#### 3.2. Physician tutor feedback

After the course, physician tutors were contacted for a face-to-face or e-mail debriefing. Of the 16 tutors, 11 responded via e-mail and two gave direct feedback. Three tutors did not participate in the debriefing. The tutors were invited to give their opinions on what went well and what could be improved about the seminar and their written and oral responses were collected, summarised and presented to the management group by AP. The tutors’ feedback was very positive overall. Many tutors enjoyed the interaction within the small group and found students to be actively involved and motivated. Some tutors suggested a more standardised seminar structure would have been beneficial. Technical issues (poor sound of videos, connection of different devices to monitors) were also mentioned as needing improvement.

We planned to also implement the expanded course format for sixth-year students in March 2020, with improvements resulting from students’ and tutors’ feedback. Due to the public health crisis with COVID-19 and the suspension of all education requiring presence of students at the University, the implementation has been postponed to next term. 

## 4. Discussion

This article describes the expansion of a simulation-based communication skills training by adding a video-reflection homework task followed by a seminar on the annotated videos. The expanded course format was successfully implemented in Autumn 2019 and was well received by students and tutors. 

Interestingly, students felt that it was more beneficial to view and annotate videos of peers than their own videos. The objective of video reflection in communication skills training is typically focused on having the learner “look at himself from a distance” and thus to support “positive self-modelling” [13]. Following this framework, we expected that students would benefit most from viewing their own videos. However, to be confronted with oneself on video may simply be uncomfortable for students. Additionally, having already received in-depth feedback by a trained SP as well as a peer may narrow the scope for self-reflection. Furthermore, learner satisfaction may not correlate with actual learning. Direct comparison of self with peer annotation could shed light on this interesting discordance.

The annotation task within this project provided little guidance to students, with the only requirement being to annotate one example of good communication skills and one example with room for improvement per video. According to a meta-analysis on video-feedback training formats, interventions including an “observation form” that structured students’ annotations had a significantly larger effect on communication skills than interventions with no such form (mean effect size of 0.55 vs. 0.21 respectively) [13]. Discussing annotated videos in small groups was generally very well received by the teaching physicians, however some would have wished for more guidance on how to structure the seminars. This is in accordance with the results from a recent qualitative study on leading video-feedback seminars, where “keeping thematic continuity of the discussion” was identified as one of the major challenges [16]. It may be that adding more structure to both the annotation task and to the seminar itself would further improve the existing programme.

Being a programme evaluation study with students’ and tutors’ perceptions as the only outcome, this project description has limitations both with regards to generalisability in other settings and to the effects on skill improvement and real-life transfer. Further studies will need to more narrowly focus on targeted research questions to allow for new insights with regards to the instructional design principles. Potential research avenues include an experimental design to directly compare self- with peer-annotation, and a qualitative study on the perception of, and ambivalence about, self-reflection in video training.

## 5. Conclusion

An expanded communication skills course with video-annotations and group discussions with a physician tutor was implemented, with the aim of addressing identified areas for improvement. The expanded course format was shown to be feasible, was well-received by students and faculty, and will now also be implemented in the sixth student year.

## Authors

The authors Anina Pless and Roman Hari share the first authorship

## Acknowledgements

We would like to thank Prof. Michael Harris for the proof-reading and revision of this manuscript. 

## Profiles

**Name of location: **University of Bern

**Field of study/professional group: **Faculty of Medicine

**Number of learners per year:** approx. 340 students since 2018.

**Is a longitudinal communication curriculum implemented?** Yes 

**In which semesters are communication and social skills taught? **Compulsory courses in the 1^st^, 2^nd^, 5^th^, 6^th^, 7^th^, and 12^th^ semesters as well as additional optional courses.

**What teaching formats are used?** Practical courses in small groups at the bedside (clinical skills training), practical courses with simulated patients (SPs) and SP feedback, lectures, seminars with video-based feedback, individual reflection based on video recordings, formative assessment with SP and tutor feedback.

**In which semesters are communicative and social competences assessed (formative or pass-relevant and/or graded)?** Formative OSCE at the end of the 2nd semester and at the end of the 5th semester, summative OSCE at the end of the 6th and 10th semesters, summative OSCE examination as part of the Federal Final Exam in Human Medicine at the end of the degree programme

**Which examination formats are used?** Summative and formative OSCE

**Who (e.g. clinic, institution) is entrusted with the development and implementation?** Steering group "Communication" of the University of Bern; a permanent interdisciplinary working group led by the Institute of Family Medicine of Bern (BIHAM) and the Institute of Medical Teaching (IML) with representatives from various clinics of the University Hospital of Bern.

## Current professional roles of the authors

- Anina Pless works as a scientific collaborator in the department of education at the Institute of Primary Healthcare of Bern, Switzerland, where she is responsible for the field „teaching communication“. She also works clinically at the medical policlinic of the University Hospital of Zurich, Switzerland.
- Roman Hari is head of the department of education at the Institute of Primary Healthcare, University of Bern, Switzerland. Ha also works as a general practitioner in Burgdorf, Switzerland.
- Beate Brem works as a scientific collaborator at the Department for Education and Media of the Institute for Medical Education, University of Bern in Switzerland. She is head of the group working with simulated participants.
- Ulrich Woermann works as a scientific collaborator at the Department for Education and Media of the Institute for Medical Education, University of Bern, Switzerland. He is head of the Learning Media unit.
- Kai Schnabel is head of the department for Education and Media of the Institute for Medical Education, University of Bern in Switzerland.

## Competing interests

The authors declare that they have no competing interests. 

## Supplementary Material

Detailed list of questions
