# Supplementary material for: Using self and peer video annotations of simulated patient encounters in communication training to facilitate the reflection of communication skills: an implementation study
Source: GMS J Med Educ. 2021 Mar 15;38(3):Doc55. doi: 10.3205/zma001451 (PMC7994885; doi:10.3205/zma001451)
Supplement: Detailed list of questions [file JME-38-3-55-s-001.pdf]

Attachment 1: Detailed list of questions

**Preparation**
Expenditure of time (0-1000min)<br>• How many minutes did you invest to prepare for the communication training?<br>• How many minutes did you spend preparing with the script?<br>• How many minutes did you spend preparing with Doccom?<br>Open questions<br>• If you worked with the script, how was this helpful in preparing the course?<br>• If you worked with Doccom, how was this helpful in preparing the course?<br>• Have there been other means of preparation, that you found helpful? If yes, what was that?
**Annotation**
Likert scale (strongly agree, agree, neither agree nor disagree, disagree, strongly disagree)<br>• The annotation task was clear and understandable to me<br>• Annotating the videos in ILIAS was feasible to me<br>• It was clear to me who I would share my video with and whose video I would annotate<br>• I benefited from annotating my own video<br>• I benefited from annotating the video of a colleague<br>• It was okay for me to be judged by a colleague<br>Expenditure of time (0-300min)<br>• How much time did you spend annotating the videos?
**Seminar**
Likert scale (strongly agree, agree, neither agree nor disagree, disagree, strongly disagree)<br>• The preparatory task in the small group immediately before the seminar was clear<br>• The time for preparation in the small group immediately before the seminar was sufficient<br>• I learned a lot by discussing the annotated video sequences<br>• The tutor's feedback and comments were instructive to me<br>• I was able to apply what I had learned in the seminar in the second communication training<br>Expenditure of time (too long – ideal – too short)<br>• Time for the seminar with the tutor was<br>Open questions<br>• What did you learn in the seminar?<br>• What did you particularly like about the seminar and should be kept?<br>• How can we improve the seminar?<br>Overall mark (1-6)<br>• I give the seminar the following grade (6=very good, 1=very bad)
